# Clustering of multiple lifestyle behaviors among migrant, left-behind and local adolescents in China: a cross-sectional study

**DOI:** 10.1186/s12889-021-10584-4

**Published:** 2021-03-19

**Authors:** Li He, Xiaoyan Li, Weidong Wang, Youfa Wang, Haiyan Qu, Yang Zhao, Danhua Lin

**Affiliations:** 1 College of Physical Education and Sports, Beijing Normal University, Beijing, China; 2 Institute of Developmental Psychology, Beijing Normal University, Beijing, China; 3 School of Sociology and Population Studies, Renmin University of China, Beijing, China; 4 Global Health Institute, School of Public Health, Xi’an Jiaotong University, Xi’an, China; 5 Department of Health Services Administration, University of Alabama at Birmingham, Birmingham, USA; 6 The George Institute for Global Health at Peking University Health Science Centre, Beijing, China; 7 WHO Collaborating Centre on Implementation Research for Prevention & Control of NCDs, Melbourne, VIC Australia

**Keywords:** Clustering, Lifestyle behavior, Migration, Left-behind, Adolescents

## Abstract

**Background:**

Influence of migration on externalized behavioral problems (e.g., aggressive) among adolescents has been well assessed, yet lifestyle behaviors of migrant, left-behind and local adolescents have been largely overlooked by researchers and policy-makers. Therefore, this study aimed to identify clustering of multiple lifestyle behaviors and their associations with migrant status among Chinese adolescents.

**Methods:**

A cross-sectional survey was conducted in 2015 in Beijing, and Wuhu city (Anhui province). Adolescents self-reported age, gender, family economic status, migrant situation, and lifestyle behaviors (i.e., physical activity, screen time, sleep, smoke, soft-drink, alcohol, fruit and vegetable consumption) via a battery of validated questionnaires. Latent class analysis was conducted to identify behavioral clusters using Mplus 7.1. ANOVA, and multivariable logistic regression were used to examine associations between migrant situations and behavioral clusters using SPSS 22.

**Results:**

Three distinct behavioral clusters were exhibited among 1364 students (mean age: 13.41 ± 0.84 years): “low risk” (*N* = 847), “moderate risk” (*N* = 412) and “high risk” (*N* = 105). The “high-risk” cluster had the highest prevalence of adolescents not meeting healthy behavioral recommendations. There were no significant differences in the prevalence of high-risk lifestyle among migrant, left-behind, rural local and urban local adolescents. But migrant adolescents had the lowest prevalence of low-risk lifestyle, followed by left-behind, rural and urban local adolescents. Moreover, compared with urban local, migrant (OR = 2.72, 95%CI: 1.88,3.94), left-behind (OR = 2.28, 95%CI: 1.46, 3.55), and rural local (OR = 1.76, 95%CI:1.03,3.01) adolescents had a higher risk of moderate-risk lifestyle.

**Conclusions:**

Clustering of assessed lifestyle behaviors differed by the migrant status. Particularly, migrant and left-behind adolescents were more likely to have moderate-risk lifestyle compared with their counterparts. Interventions that promote moderate to vigorous physical activity and consumption of fruits and vegetables simultaneously are needed among them.

**Supplementary Information:**

The online version contains supplementary material available at 10.1186/s12889-021-10584-4.

## Background

In China, with the steady increase of rural-to-urban migrant laborer over the past decades, a growing number of rural children and adolescents has been either left at home by one or both parent(s) who cannot afford to bring their families to the cities where they work (left-behind) or relocated to urban areas with their parents (migrant). In 2015, the amount of migrant and left-behind children and adolescents at compulsory education stage has reached 33.86 million, with close to 20.19 million left-behind in rural areas [1]. Similar to their migrant parents, migrant and left-behind children and adolescents are typically socially disadvantaged groups that have been attracted great research interests in the impact of migration on individuals, families, and society in the literature. However, lifestyle behaviors of migrant and left-behind children and adolescents have been largely overlooked by researchers and policy-makers [2].

According to family systems theory [3], parents are known to play an important role in the establishment of a child’s secure attachment relationships, for emotional support, self-regulation, as well as the development of social skills and lifestyle habits [4]. Previously, abundant literature has suggested that the migration status of Chinese children or adolescents and their parents was detrimental to their academic achievements, nutrition, body weight, and mental health. Compared to those adolescents with non-migrant parents, left-behind children or adolescents have had poorer nutritional outcomes, a higher risk of stunting, depression, or anxiety, more self-injuries, and a marginally higher risk of substance use including alcohol, smoking, and any substance use, when they are separated from their parents at a younger age and for a longer time [4, 5]. Similarly, rural-to-urban migrant children or adolescents in China are more vulnerable to mental health problems than local children, including social and separation anxiety, depression, loneliness and low self-esteem [6–8]. In the respect of behavioral health, significant differences have been observed in behavioral problems among migrant, left-behind and local children aged 3–16 years [2, 4]. However, they focused on the internalizing problems (i.e., emotional disturbances including schizoid or depressed, social-withdrawal and somatic complaints) and externalizing behavioral problems (refer to behavioral deficits, such as aggressive, delinquent, etc.). There were no studies so far clustering multiple lifestyle behaviors among migrant, left-behind, urban and rural local children or adolescents, and examining the associations of migrant status with lifestyle behavior clusters.

Adolescence is often considered as an important stage of life. Lifestyle behavioral habits, including physical activity (PA), sedentary behavior (SB), sleep, drink, and diet, are commonly established at this stage and tend to track throughout adulthood [9, 10]. Since research has shown that lifestyle behaviors do not occur in isolation, and combinations of multiple behaviors may contribute more to health or disease prevention than single behavior [11–14], identifying clustering patterns of multiple behaviors among adolescents should be given more public health consideration in order to gain the greatest health benefits among migrant, left-behind, urban and rural local adolescents.

Although there has been a growing number of studies investigating the clustering or cooccurrence of multiple modifiable behaviors among adolescents, few of them were conducted in China [14–23]. Parker et al. [14] systematically reviewed studies published up to May 2018, and identified 36 studies examining clusters of activity-related behaviors among adolescents, as well as socio-demographic characteristics and the modifiable correlates associated with behaviors. The most commonly reported combinations of behaviors are PA and SB (13 studies); or PA, SB and diet (13 studies). The remaining 10 studies investigating the cluster of PA, SB and risk-related behaviors such as smoke and alcohol consumption, but they were typically comprised of older adolescents. Moreover, few studies were found including sleep-related factors into their cluster analysis. As insufficient sleep is a risk factor of chronic diseases [24], sleep duration is crucial for adolescents’ health and should be considered. Chen et al. recently has investigated age and gender difference in the co-existence of PA and SB among 10 to 18 years children and adolescents in Shanghai [15]. However, this study disregarded sleep, diet and other risk-related behaviors such as smoke and drink. Collectively, there were no research examining clustering patterns of multiple lifestyle behaviors among migrant, left-behind, urban and rural local adolescents in China and other countries.

To address this research gap, this study aims to examine behavior clusters of abovementioned four groups of adolescents in rural and urban China, and investigate associations between migration status and behavioral clusters. Understanding patterns of behaviors among adolescents with different migrant statuses will help to identify at-risk individuals, inform specific lifestyle behavior change, and further eliminate health inequalities and disparities in the current society.

## Method

### Data source

Cross-sectional data from the Survey on Child and Adolescent Health Development in China (year 2014–2015) were used for this study. A total of 1402 young adolescents in their first year of middle school were recruited through convenience sampling from eight public middle schools in Beijing City (schools, *N* = 4; students, *N* = 775) and Wuhu city (Anhui Province; schools, *N* = 4; students, *N* = 627). Three suburban schools and one urban school were selected from a district in the west and northwest of Beijing. This district has 3.69 million residents, accounting for 17.0% of the resident population in Beijing in 2015 [25, 26]. Among the four schools, one was a district-level demonstrative school, and others were ordinary schools ranked at low-middle level in this district. Other four schools were selected from towns (Schools, *N* = 2) and villages (Schools, *N* = 2) in a county in the southwest of Wuhu. In 2015, the residents of this county accounted for 28.0% of the total residents in Wuhu [27, 28]. All the four rural schools ranked similar in the county at the middle-level. We received written informed consent from at least one of parent or guardian for all students participating in the survey. Participants completed questionnaire during class time under the supervision of trained researchers and class teachers. The final sample included 1364 participants for analysis after removing individuals with over five missing lifestyle behavior variables (*N* = 38). Study procedures were approved by the Beijing Normal University Ethical Advisory Committee.

### Measurements

The questionnaire used in the survey included several subscales and questions to assess general background of children and their parents (age, education level, income, migration, etc.), personal characteristics (individual perception of weight, body shape and lifestyle, etc.), and information on friends, family, school and community. Questions on lifestyle behaviors referred to the Global Youth Risk Behavior Survey which had a substantial or higher reliability in previous studies (kappa = 61–100%) [29]. More psychometric properties of the questionnaire were described in previous studies [29–32].

### Social demographic variables

The general part of the questionnaire included questions on the adolescents’ age, sex, migrant background, and socioeconomic status (SES). Adolescents were asked to rate the SES of their family using 5-point Likert scale from “1 = very poor” to “5 = very rich”. The location of adolescents’ or their parents’ “hukou” registration was reported by adolescents. Also, adolescents self-reported their hukou type (i.e., rural or urban hukou). The eligibility criteria for rural-to-urban migrant children included the following: (1) location of hukou registered outside of Beijing, (2) type of hukou is rural (i.e., agricultural hukou), and (3) now living with their rural-to urban migrant parent(s) who have migrated to Beijing for employment at least 6 months [1]. To be consistent with the national population census in China, the rural left-behind adolescents in the present study referred to adolescents who lived in the rural hometown, had rural hukou, and have been separated from one or both of their parents (migrated outside of the county/city/province to work) for at least 6 months [1]. In total, we distinguished four groups in our sample: migrant rural to urban (MA, *N* = 421), rural left-behind (LBA, *N* = 432), urban local (ULA, *N* = 351), and rural local adolescents (RLA, *N* = 160, whose parents did not migrate outside of the county/city/province to work). Height and weight were measured objectively for calculating their body mass index (BMI). All participants were required to wear light clothing with no shoes. Continuous BMI values were included in the analysis.

### Lifestyle behaviors

Health-related lifestyle behaviors included fruit and vegetable consumption, beverage and alcohol drinking, playing computer games, PA, smoking, and sleeping.

#### Fruit and vegetable consumption (eating behaviors)

Adolescents were asked to report their intake frequency of fruits (except juice), vegetables and soft drinks over a one-week period prior to the survey with three questions. For example, “During the past week, how many times did you had fruits (except juice)?” Or “how many bottle/cans/cups of sparkling water you drank during the past week?”. For each question, we created a binary variable indicating whether the daily intake of fruits, vegetables and soft drinks met healthy guidelines (meet coded as 1/Not meet coded as 0). By the nutrition guideline in China, adolescents are recommended to eat vegetables (averagely, 300 ~ 500 g/day) each meal, and eat fruits (averagely 200 ~ 350 g/day) every day [33]. Therefore, adolescents with eating fruits 7 times or more in the past week were coded as meeting the guideline of eating fruits. Adolescents with a frequency of consuming vegetables 21 times or more in the past week were grouped as meeting the guideline of eating vegetables. In addition, the glasses/cups/bottles of consumption of soft drinks was split at any or non to create a drinking or not drinking category (drink coded as 0/not drink coded as 1). This category was also based on the recommendation of nutrition guideline for Chinese (not drink soft-drinks and alcohol) and was consistent with previous studies [16, 33, 34].

#### Moderate to vigorous physical activity (MVPA)

During the past week, the number of days per week participating in MVPA for at least 60 min (referring to the physical activities that can speed up the heart rate and make adolescents breathe hard) were obtained from the adolescents. For the analysis, participants were grouped into those that met the guidelines (coded as 1) and those that did not meet the guidelines (coded as 0) based on the threshold value for MVPA of 7 days per week according to the PA guidelines issued by the World Health Organization [35].

#### Screen time

Time spent on screen-based activities was assessed by the following question: “On average, how long do you spend on computer games or things unrelated to learning (e.g., watching movies, surfing internet, shopping, and online chat) every day?” The response alternatives were weekdays: _hours_minutes and weekends: _hours_minutes. Considering that adolescents aged 12–17 years old are recommended to limit their screen time (TV, computer, inactive video game playing) to no more than 2 h per day [36], a 2-h cut-off was used as most recommendations restrict screen-based activities to approximately 2 h per day, and this cut-off has also been used in previous relevant studies [37, 38]. Moreover, considering that a previous analysis showed a significant difference in screen time between weekdays and weekends [28], for the present research question, the 2-h screen time per day threshold was used to classify participants into not meeting (> 2 h/day, coded as 0) and meeting (≤ 2 h/day, coded as 1) screen time groups for weekdays and weekends separately.

#### Sleep behavior

Self-reported sleep duration was collected via the following question: “On a weekday/weekend, how many hours do you sleep every night?” Sleep time was collected in hours and minutes for weekdays and weekends. Given that previous studies showed significant sleep duration differences between weekdays and weekends [39, 40], both weekday and weekend sleep duration were included in the analyses in the present study. For teenagers, 8 to 10 h was considered appropriate [41]. Finally, categorized measures of sleep duration (appropriate sleep coded as 1, not appropriate coded as 0) were included into the analyses.

#### Smoking and drinking behaviors

Adolescents reported their smoking and alcohol drinking behaviors in response to two questions: “Have you ever smoked? If smoked, please record the times of smoking in the past week.” and “Have you ever had a drink (beer, red wine or white wine)? If drank, please record the times of drinking in the past week”. Responses were categorized into no if they did not smoke or drink during the past week (coded as 1), and yes if they smoked or drank over one time during the past week (coded as 0).

### Statistical analysis

A latent class analysis was performed with the above ten lifestyle indexes as observation variables using Mplus Version 7.1. Ratio difference test was used to test the differences in clusters among adolescents with different migrant status. The analysis of variance (ANOVA) was conducted to examine differences in the ten lifestyle variables by clusters. And then the Least-Significant Difference (LSD) analysis was used for the post-hoc test if ANOVA showed significant main effects. To further examine influences of migrant status on behavioral clusters, multiple logistic regression analysis was performed, with clusters of lifestyle behaviors as the dependent variable, migrant status as the independent variable, adjusting for age, gender, family economic status and body mass index as covariates. Odds rations (OR) and 95% confidence intervals (CI) were used to quantify the strength of correlations. All the ANOVA and multiple logistic regression were performed via IBM SPSS Version 22 (Armonk, NY: IBM Corp.). The statistical significance for all tests was established at *p* < 0.05.

## Results

### Descriptive statistics and variable correlations

As shown in Table 1, a total of 1364 adolescents with a mean age of 13.41 ± 0.84 years were enrolled including 755 male (56.30%) adolescents, 432 left-behind (31.70%), 160 rural local (11.70%), 421 rural-to-urban (30.90%), and 351 urban local adolescents (25.70%). With respect to the ten lifestyle behaviors, the frequencies of not meeting behavioral guidelines ranged from 3.00% (smoking) to 83.87% (MVPA) among the overall sample. Moreover, the frequencies of not meeting the guidelines ranged from 3.94% (smoking) to 90.05% (MVPA), 5.00% (smoking) to 88.13% (MVPA), from 3.10% (smoking) to 82.42% (MVPA), and from 0.85% (smoking) to 76.07% (beverage drinking) among left-behind, rural local, migrant, and urban local adolescents, respectively. The correlation matrix showed most of the correlations of variables were lower than ±0.3 (Additional file 1: Table 1). Moreover, the variance inflation factors were all less than 10 (range: 1.00–1.15), suggesting that the association was unlikely to influence the clustering procedure.

**Table 1:** Demographical characteristics of Chinese adolescents: Survey on child and adolescent health development in China (Year 2014–2015) (*N* = 1364)

Characteristics	Total	left-behind adolescents	migrant adolescents	rural local adolescents	Urban local adolescents
Population (N, %)	1364 (100.00)	432 (31.70)	421 (30.90)	160 (11.70)	351 (25.70)
Age (years, M, SD)	13.41 (0.84)	14.08 (0.71)	12.99 (0.69)	13.89 (0.79)	12.87 (0.35)
Gender (N, %) ^a^					
Girls	585 (43.70)	179 (41.44)	157 (37.29)	68 (42.50)	181 (51.57)
Boys	755 (56.30)	248 (57.41)	256 (60.81)	86 (53.75)	165 (47.00)
Family economic status (M, SD)	3.61(.079)	3.37 (0.67)	3.66 (0.75)	3.24 (0.79)	3.99 (0.78)
Body mass index (M, SD)	20.05 (3.94)	19.21 (3.31)	20.71 (4.13)	19.06 (3.32)	20.65 (3.90)
**Lifestyle behaviors (not meeting guidelines) (n, %)**					
FE (< 7 times/week; *N* = 1296)	617 (45.23)	191 (44.21)	239 (56.77)	61 (38.13)	126 (35.90)
VE (< 21 times/week; *N* = 1264)	1022 (74.93)	329 (76.16)	316 (75.06)	115 (71.88)	262 (74.64)
BD (≥1 glasses/week; *N* = 1319)	741 (54.33)	271 (62.73)	237 (56.29)	92 (57.50)	141 (40.17)
MVPA (< 7 days/week; *N* = 1311)	1144 (83.80)	389 (90.05)	347 (82.42)	141 (88.13)	267 (76.07)
STD (> 2 h/day; *N* = 1303)	263 (19.28)	109 (25.23)	90 (21.38)	25 (15.63)	39 (11.11)
STE (> 2 h/per day; *N* = 1305)	628 (46.04)	205 (47.45)	224 (53.21)	72 (45.00)	127 (36.18)
SDD (< 8 h or > 10 h per day; *N* = 1315)	506 (37.10)	191 (44.21)	114 (27.08)	78 (48.75)	123 (35.04)
SDE (< 8 h or > 10 h per day; *N* = 1326)	474 (34.75)	151 (34.95)	163 (38.72)	46 (28.75)	114 (32.48)
SM (≥1time/week; *N* = 1305)	41 (3.00)	17 (3.94)	13 (3.09)	8 (5.00)	3 (0.85)
AD (≥1 times/week; *N* = 1314)	302 (22.14)	95 (21.99)	84 (19.95)	32 (20.00)	91 (25.93)

*Note.* ^a*.*^ Twenty-four adolescents missed the gender variable (1.76%; five left-behind adolescents, six rural local adolescents, eight migrant adolescents, five urban local adolescents). Family economic status was reported from 1 (very poor) to 5 (very good), the score of it was the mean score. All the ten lifestyle variables were described by the frequency of not meeting guidelines, respectively. *FE* fruit eating, *VE* vegetable eating, *BD* beverage drinking, *MVPA* moderate to vigorous physical activity, *STD* screen time (weekdays), *STE* screen time (weekends), *SDD* sleep duration (weekdays), *SDE* sleep duration (weekends), *SM* smoking, *AD* alcohol drinking, *M* mean, *N* number, *SD* standard deviation

### Latent clusters of multiple lifestyle behaviors among Chinese adolescents

Five latent models were set up, representing 1 to 5 latent clusters. The statistical fitting parameters were shown in Table 2. According to the Akaike information criterion (AIC), adjusted Bayesian information criterion (aBIC), the likelihood ratio test (LMR) value, entropy, and the suggestion that the number of participants in the least cluster should be no less than 5%, the model with four or five clusters was not better than the model with three clusters. Judging from the synthesis of various fitting parameters and the theoretical significance and interpretability of the results, it seemed that the model with three clusters was more suitable than the others. For the three-cluster model, the cluster probabilities were 62.10% (*n* = 847, cluster 1), 30.20% (*n* = 412, cluster 2) and 7.70% (*n* = 105, cluster 3). The three-cluster model was relatively suitable for determining comprehensive clusters in terms of practical significance, model simplicity and various fitting parameters.

**Table 2:** Index of latent classes of multiple lifestyle behaviors: Survey on child and adolescent health development in China (Year 2014–2015) (*N* = 1364)

Class	k	G^**2**^/LL	df	χ^**2**^	AIC	BIC	aBIC	Entropy	LMR	BLRT	Probabilities
1	10	898.58	1025	2283.87	14,279.64	14,331.84	14,300.08				1.000
2	21	659.88	976	1187.43	13,863.59	13,973.22	13,906.51	0.616	< 0.0001	< 0.0001	0.644/0.356
**3** ^a*.*^	**32**	**595.86**	**969**	**1125.13**	**13,786.73**	**13,953.78**	**13,852.13**	**0.688**	**< 0.01**	**< 0.0001**	**0.078/0.301/0.621**
4	43	530.44	958	1086.72	13,742.33	13,966.81	13,830.21	0.588	0.1079	< 0.0001	0.072/0.223/0.500/0.206
5	54	484.87	947	926.48	13,717.77	13,999.67	13,828.13	0.635	0.3604	< 0.0001	0.153/0.096/0.112/0.609/0.030

*Note.*^a*.*^ Bold indicates the final choice for the number of clusters. *AIC* Akaike information criterion, *BIC* Bayesian information criterion, *aBIC* adjusted Bayesian information criterion. Entropy indicates classification quality. LMR = *P* value of likelihood ratio test. BLRT = *P* value of bootstrap likelihood ratio test

**Fig. 1:**
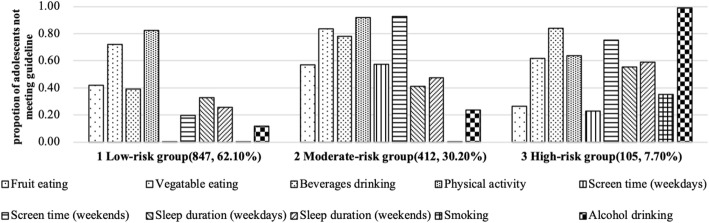
Clusters of multiple lifestyle behaviors among Chinese adolescents: Survey on child and adolescent health development in China (Year 2014–2015)

Figure 1 showed the percentage of adolescents not meeting the guidelines on the ten lifestyle behaviors in each cluster. Mean differences in the main variables among latent clusters was shown in Table 3. According to the descriptive statistics characteristics of the three latent clusters and the results of Pos hoc, the present study named the three clusters as: (1) The low-risk group (Cluster 1, *n* = 847), having the smallest proportion of not meeting the guidelines for lifestyle behaviors: with no smoking, short screen time on weekdays, the least alcohol drinking and the most amount fruit eating and vegetable eating. (2) The moderate-risk group (Cluster 2, *n* = 412), which was relatively less serious, with a lower proportion of adolescents not meeting the behavioral guidelines: less MVPA, spending more screen time, eating less vegetables. (3) The high-risk group (Cluster 3, *n* = 105): highest prevalence of participants not meeting the behavioral guidelines, especially for smoking and alcohol drinking.

**Table 3:** Differences in the main variables between lifestyle clusters: Survey on child and adolescent health development in China (Year 2014–2015) (*N* = 1364) ^a^

	Group 1 Low-risk (62.10%)		Group 2 Moderate-risk (30.20%)		Group 3 High-risk (7.70%)		F	η^2^	Pos hoc	Group 2 - Group 1	Group 3 - Group 1	Group 3 - Group 2
	M	SD	M	SD	M	SD				95% *CI*	95% *CI*	95% *CI*
FE	0.43	0.50	0.60	0.49	0.26	0.44	21.19^***^	0.04	2 > 1 > 3	0.14 ~ 0.34	0.02 ~ 0.21	−0.18 ~ − 0.06
VE	0.79	0.41	0.90	0.30	0.61	0.49	15.92^***^	0.04	2 > 1 > 3	0.09 ~ 0.26	0.01 ~ 0. 18	−0.13 ~ − 0.03
BD	0.42	0.49	0.79	0.41	0.84	0.37	129.36^***^	0.14	3 > 1,2 > 1	0.30 ~ 0.41	0.28 ~ 0.44	−0.08 ~ 0.10
MVPA	0.86	0.35	0.95	0.23	0.68	0.47	30.99^***^	0.04	2 > 1 > 3	0.13 ~ 0.32	−0.24 ~ −0.05	−0.12 ~ − 0.05
STD	0.00	0.07	0.58	0.49	0.25	0.44	519.24^***^	0.43	2 > 3 > 1	0.44 ~ 0.54	0.13 ~ 0.29	−0.37 ~ − 0.19
STE	0.21	0.41	0.96	0.20	0.78	0.41	612.76^***^	0.48	2 > 3 > 1	0.59 ~ 0.67	0.32 ~ 0.51	−0.32 ~ −0.13
SDD	0.34	0.47	0.42	0.49	0.61	0.49	14.50^***^	0.03	3 > 2 > 1	0.01 ~ 0.12	0.07 ~ 0.28	0.01 ~ 0.22
SDE	0.26	0.44	0.46	0.50	0.66	0.48	49.43^***^	0.07	3 > 2 > 1	0.12 ~ 0.24	0.14 ~ 0.35	0.05 ~ 0.17
SM	0.00	0.05	0.00	0.05	0.35	0.48	286.88^***^	0.30	3 > 2, 3 > 1	−0.01 ~ 0.01	0.17 ~ 0.34	0.17 ~ 0.34
AD	0.13	0.33	0.23	0.42	1.00	0.00	287.07^***^	0.31	3 > 2 > 1	0.04 ~ 0.14	0.53 ~ 0.70	0.42 ~ 0.61

*Note.* ^a.^ In the results of Pos hoc, the risk of high-risk group was more severe due to smoking and alcohol drinking problems compared to the moderate-risk group, though the high-risk group counted three times as the highest score which was same as the moderate-risk group. ^***^p < 0.001. The Least-Significant Difference (LSD) analysis was used for the post-hoc test. 95% CI represents 95% confidence interval. *FE* fruit eating, *VE* vegetable eating, *BD* beverage drinking, *MVPA* moderate to vigorous physical activity, *STD* screen time (weekdays), *STE* screen time (weekends), *SDD* sleep duration (weekdays), *SDE* sleep duration (weekends), *SM* smoking, *AD* alcohol drinking

### Disparities in latent clusters of lifestyle behaviors between four group adolescents

**Fig. 2:**
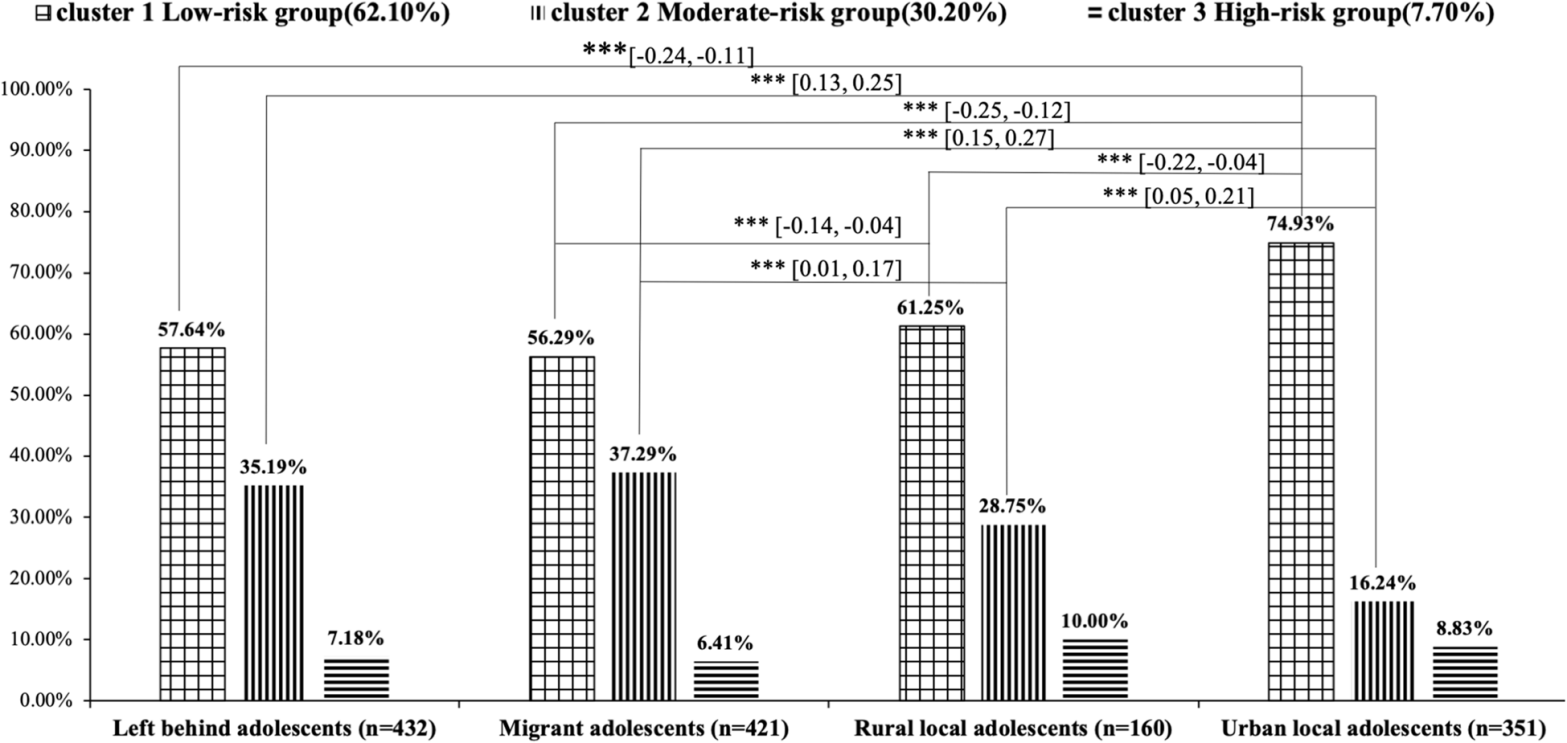
Proportions of lifestyle clusters by migration status among adolescents: Survey on child and adolescent health development in China (Year 2014–2015)

As shown in Fig. 2, for the moderate risk cluster, the ratio of MA (37.29%) was higher than LBA (35.19%), RLA (28.75%) and ULA (16.24%). There were significant differences in the ratio of MA, RLA and ULA, as well as the ratio of LBA and ULA (Zs > Z_0.001_). For the low-risk cluster, the ratio of MA (56.29%) was the lowest, followed by the ratio of LBA (57.64%), RLA (61.25%), and ULA (74.93%). Based on the Post Hoc comparison, significant differences were observed between the ratio of ULA and other three groups (Zs > Z_0.001_). In addition, there was no significant difference in the ratios of participants in the high-risk group (cluster 3) among the four groups.

### Associations of migrant situations and clusters of multiple lifestyle behaviors

As shown in Table 4, the model 1 was individually tested in separate logistic regression analyses with only age, gender, SES and BMI. Using low-risk cluster as the reference, the results indicated that the probabilities of elder adolescents entering the moderate-risk group (OR = 1.27, 95% CI [1.00, 1.48]) and high-risk group (OR = 1.34, 95% CI [1.04, 1.74]) were higher than young adolescents. Compared to female adolescents, males were more likely to exhibit high-risk behavior (OR = 2.67, 95% CI [1.64, 4.35]). In the model 2, after controlling for the age, gender, SES, and BMI, migrant (OR = 2.72, 95% CI [1.88, 3.94]), left-behind (OR = 2.28, 95% CI [1.46, 3.55]) and rural local adolescents (OR = 1.76,96%CI [1.03,3.01]) were more likely to have moderate-risk lifestyle cluster, compared with urban local adolescents.

**Table 4:** Multiple logistic regression model for the latent clusters of lifestyle behaviors: Survey on child and adolescent health development in China (Year 2014–2015)

	Model 1				Model 2			
	Moderate-risk vs Low-risk cluster		High-risk vs Low-risk cluster		Moderate-risk vs Low-risk cluster		High-risk vs Low-risk cluster	
	*OR*	95% CI	*OR*	95% CI	*OR*	95% CI	*OR*	95% CI
Age	1.27*	1.09–1.48	1.34*	1.04–1.74	1.18	0.97–1.43	1.47*	1.05–2.05
Gender (ref. girls)	1.28	0.99–1.65	2.67**	1.64–4.35	1.19	0.92–1.53	2.71**	1.66–4.42
Family economic status	0.92	0.78–1.08	1.20	0.90–1.59	0.99	0.84–1.18	1.16	0.87–1.55
Body mass index	1.03	0.99–1.06	1.00	0.95–1.06	1.03	0.99–1.06	1.00	0.94–1.06
Left-behind (ref. Urban local)					2.28**	1.46–3.55	0.68	0.33–1.42
Migrant (ref. Urban local)					2.72**	1.88–3.94	0.82	0.45–1.48
Rural local (ref. Urban local)					1.76*	1.03–3.01	0.83	0.36–1.94

*Note*. * *p* < 0.05, * *p < 0.001. *OR* odds ratio, *CI* confidence interval

## Discussion

The present study firstly compares the group differences in clustering of multiple lifestyle behaviors among migrant, left-behind, rural local and urban local adolescents in China. Findings about how various behaviors blend together to form “overall” lifestyle provides a basis to develop tailored interventions that promote effective and sustainable lifestyle change among this understudied population. Consistent with previous literature, the present study revealed that adolescents engaged in a variety of lifestyle behaviors, and the cluster patterns were a complex mix of healthy and unhealthy behavioral patterns [14, 18]. All clusters consisted of at least two unhealthy behaviors in current study, which implied that an overall healthy lifestyle was not observed among participants. Therefore, interventions for changing at least two risk lifestyle behaviors simultaneously are needed for adolescents.

Three distinguished behavioral patterns ranging from high risk to low risk were exhibited among young adolescents, with a higher prevalence of adolescents (62%) having a relatively low-risk lifestyle pattern. The low-risk cluster was characterized by nonsmokers, the lowest levels of screen time, beverage and alcohol consumption, along with a moderate level of fruits’ and vegetable’ consumption and MVPA. There was no high diet quality/ low sedentary behavior/high physical activity pattern observed previously. In contrary, a low diet quality/high sedentary behavior pattern, or the cluster of high physical activity and low sedentary behavior was prevalent among adolescent in US, UK, Dutch, Span and other European countries [14, 18]. For example, in a large cross-sectional study of 2084 adolescents from 10 European countries, where Ottevaere et al. identified the largest proportion of adolescents (42%) was categorized as inactive but with low levels of sedentary behavior and a high diet quality [42]. In Brazilian adolescents, two mostly healthy clusters were also characterized by low levels of sedentary behavior and low consumption of unhealthy food, and the highest levels of physical activity and adequate diet [20]. The present study contributed a unique finding to the literature of lifestyle clusters. Combined with previous studies, these findings suggest that low-risk (or healthier) cluster patterns might be related to the culture of particular countries, which implies that different strategies to promote lifestyle are required under the different social context.

In the high-and moderate-risk clusters, insufficient MVPA and fruit and vegetable consumption were common concerns in the present study. Adolescents in the moderate-risk cluster did not report smoking, but reported the highest level of screen time throughout the week and the lowest level of MVPA and fruit and vegetable consumption, as well as having moderate levels of sleep and alcohol and beverage drinking. The recommendation for Chinese adolescents is one-piece fruit and three times vegetable each day. However, approximately 60–90% of the adolescents in the moderate-risk cluster reported fewer fruit intake (< 7 times/week) and vegetable intake (< 21 times/week). This implies a critical need to promote fruit and vegetable consumption among young adolescents. In previous studies, adolescents in the unhealthier clusters in Western countries also failed to meet guidelines for one or more health behavior(s); for example, high levels of screen time, low fruit and vegetable consumption, and inactivity tend to cluster in this age group [16, 19, 23]. Although there were differences in the numbers of health-related behaviors, measurements, analytical approaches and countries between the current study and other studies of adolescents, they consistently suggest that behavioral interventions to promote healthy behaviors are challenging, and more studies are required to promote a healthy lifestyle in adolescent population.

Importantly, for the impact of migration on behavioral cluster, the present study found that migrant adolescents had the lowest prevalence of low-risk lifestyle patterns, followed by left-behind, rural local, and urban local adolescent. Moreover, migrant and left-behind adolescents had a significantly higher prevalence of moderate-risk lifestyle than the rural and urban local adolescents. This may partly reveal the potential impact of migration on children. Migration means a change of original living surroundings and adaption to new environments for adolescents. For adolescents who move from rural areas to urban areas with their parents, although they can live with one or both parent(s), adapting to a new school, social and physical environment can be challenging. As for the left-behind adolescents, although they have no difficulties in dealing with a new social environment, living without one or both parent(s) may lead to less parental care or supervision, so as to leading to the emergence of behavioral problems. Accordingly, to promote healthy eating, reducing levels of insufficient physical activities, and screen time, so as to achieve the target of a 15% relative reduction in insufficient physical activity among children by 2030, more attention should be paid on the migrant and left-behind adolescents [43]. As previous studies suggest that disadvantaged parents may have lower health literacy, and are unclear about their own or their child’s health risks, so they may not be able to recognize negative changes in their child’s emotional health and lifestyle and provide timely support to their children [44, 45]. Health education about the importance of fruits and vegetables, exercise and reduced screen time, as well as the behavioral guidelines for health could be provided to migrant families or guardian stayed with the left-behind and rural local adolescents.

However, different from our hypothesis, the prevalence of rural local adolescents who had high-risk behavioral pattern is slightly higher than migrant, left-behind, and urban adolescents, but there were no significant differences in four groups of adolescents. High-risk behavioral cluster included multiple risk behaviors (e.g., low levels of vegetable consumption, high levels of screen time on weekends, short sleep time throughout the week, smoking, drinking beverages, and alcohol) in current study. The non-significant difference between four groups might be understandable because of the small number of adolescents who had high-risk behavioral cluster. Only 7.70% of adolescents in our study exhibited the high-risk behavioral cluster. To advance our knowledge about the non-significant difference in high-risk behavioral cluster, we urgently need large-scale representative surveys to collect comprehensive and longitudinal information about children’s behaviors and their multilevel correlates to identify behavioral patterns among rural local, left-behind, migrant and urban local adolescents in order to better help migrant and non-migrant families nurture thriving youth in rural China.

### Limitations and strengths

The present study makes a unique contribution to the research of lifestyle patterns by examining the impact of migration on children’s multiple lifestyle behavior. It is one of the first in investigating the clustering of dietary (including fruits and vegetables consumption, alcohol, and soft-drink), PA, screen time and smoke among this understudied population. The utilization of latent cluster analysis appears to be a meaningful and useful technique that advances our understanding of health-related adolescent behavior. Although findings could imply that which behaviors and which adolescent groups need more attention, several limitations should be noted:

First, participants were recruited only from two cities (Beijing vs. Wuhu) in China and hence cannot be nationally representative, which limits the generalizability of the findings. It could be interesting for future studies to examine the stability of clusters in a large national and representative sample. Second, this study uses a cross-sectional design, which provides evidence for associations but not for causation. Third, no specific amount of foods was recorded. Only the frequency of weekly consumption of different food groups was self-reported, which may inaccurately report adolescents meeting nutrition guideline. However, it would be difficult to use more accurate methods in population-based epidemiological research, such as direct observation, to provide data regarding specific grams of fruits and vegetables consumed. We were also unable to assess the adolescents’ behaviors objectively using smartphone or ecological momentary assessment or other real-time monitoring methods, because students were not allowed to carry mobile phones at school, and lack of accelerometers could be applied all study participants. Finally, different from other studies, measures of other screen time, such as watching TV, using computer to do homework, cellphone use, were not available in the present dataset [12, 20]. This may cause the difference between our findings and other studies. Given some studies have shown that correlates of different types of sedentary behavior may be different [46], future research should examine these screen time alongside those investigated in the present study as a means of further analyzing the difference in composition of screen time by the migrant status, in order to guiding intervention efforts.

## Conclusion

In summary, our study concludes that adolescents have multifaceted lives that largely represent a low-risk lifestyle. A single overall healthy lifestyle does not exist in adolescent participants; instead, unhealthy behaviors are usually concomitant, which implies that behavioral interventions are needed and full of challenges. Changing at least two unhealthy behaviors simultaneously is required for adolescents. The clustering of assessed lifestyle behaviors differed by the participants’ migrant status and their parents’ migrant status. This suggests the potential need for different behavioral intervention targets to reduce risky behaviors in subgroups of adolescents. Particularly, create strategies to promote moderate to vigorous physical activity, and consumption of fruits and vegetables simultaneously in migrant and left-behind adolescents is urgent. There is a need for longer follow-up studies on the behavioral trends of migrant, left-behind and rural local adolescents, and to examine modifiable determinants of different lifestyles among the migrant, left-behind, urban local and rural local adolescents in China, respectively.

## Supplementary Information

**Additional file 1: Table 1.** Correlation matrix for lifestyle behaviors, gender and age (*N* = 1364) ^*a*^.

## Data Availability

The datasets used and/or analyzed during the current study are available from the corresponding author on reasonable request.

## Abbreviations

AIC: Akaike information criterion
aBIC: Adjusted Bayesian information criterion
ANOVA: Analysis of variance
BIC: Bayesian information criterion
BLRT: Bootstrap likelihood ratio test
CI: Confidence interval
LBA: Rural left-behind adolescents
LMR: Likelihood ratio test
MA: Migrant rural to urban adolescents
MVPA: Moderate to vigorous physical activity
OR: Odds ratio
PA: Physical activity
RLA: Rural local adolescents
SES: Socioeconomic status
SB: Sedentary behavior
ULA: Urban local adolescents
