# Supplementary material for: Clustering of multiple lifestyle behaviors among migrant, left-behind and local adolescents in China: a cross-sectional study
Source: BMC Public Health. 2021 Mar 19;21:542. doi: 10.1186/s12889-021-10584-4 (PMC7980326; doi:10.1186/s12889-021-10584-4)
Supplement: Supplementary file 1 — Additional file 1: Table 1. Correlation matrix for lifestyle behaviors, gender and age (N = 1364) a. [file 12889_2021_10584_MOESM1_ESM.docx]

**Title Page:**

Clustering of multiple lifestyle behaviors among migrant, left-behind and local adolescents in China: a cross-sectional study

Li He (PhD)^1^, Xiaoyan Li (MD)^#2^, Weidong Wang (PhD)^3^, Youfa Wang (PhD)^4^, Haiyan Qu (PhD)^5^, Yang Zhao (PhD)^6,7^, Danhua Lin* (PhD)^2^

1, School of Physical Education and Sports, Beijing Normal University, China. Email: aprilhelly@bjmu.edu.cn

2, Institute of Developmental Psychology, Beijing Normal University, China. Email: xiaoyan.li@mail.bnu.edu.cn

3, School of Sociology and Population Studies, Renmin University of China, China. Email: wwd@ruc.edu.cn

4, Global Health Institute, School of Public Health, Xi’an Jiaotong University, China. Email: youfawang@gmail.com

5, Department of Health Services Administration, University of Alabama at Birmingham, USA. Email: hyqu@uab.edu

6, The George Institute for Global Health at Peking University Health Science Centre, Beijing, China. Email: zhaoyang001@hsc.pku.edu.cn

7, WHO Collaborating Centre on Implementation Research for Prevention & Control of NCDs, Victoria, Australia

***Corresponding author**: Danhua Lin, Institute of Developmental Psychology, Beijing Normal University, China. E-mail: danhualin@bnu.edu.cn

# Xiaoyan Li work as co-first author

**Additional file 1: Table 1 Correlation matrix for lifestyle behaviors, gender and age (N = 1,364)** *^a^*

| **Variable** | FE | VE | BD | MVPA | STD | STE | SDD | SDE | SM | AD | Gender | Age |
| --- | --- | --- | --- | --- | --- | --- | --- | --- | --- | --- | --- | --- |
| FE | 1 |  |  |  |  |  |  |  |  |  |  |  |
| VE | 0.21^**^ | 1 |  |  |  |  |  |  |  |  |  |  |
| BD | 0.02 | 0.05 | 1 |  |  |  |  |  |  |  |  |  |
| MVPA | 0.16^**^ | 0.10^**^ | 0.03 | 1 |  |  |  |  |  |  |  |  |
| STD | 0.02 | 0.02 | 0.19^****^ | 0.03 | 1 |  |  |  |  |  |  |  |
| STE | 0.03 | 0.05 | 0.21^**^ | 0.02 | 0.43^**^ | 1 |  |  |  |  |  |  |
| SDD | 0.00 | -0.02 | 0.06^*^ | 0.02 | 0.06^*^ | 0.06^*^ | 1 |  |  |  |  |  |
| SDE | 0.01 | -0.02 | 0.06^*^ | -0.03 | 0.15^**^ | 0.11^**^ | 0.08^**^ | 1 |  |  |  |  |
| SM | 0.01 | -0.03 | 0.07^*^ | 0.00 | 0.01 | 0.06^*^ | 0.02 | 0.04 | 1 |  |  |  |
| AD | 0.00 | -0.04 | 0.11^**^ | -0.03 | 0.12^**^ | 0.17^**^ | 0.09^**^ | 0.11^**^ | 0.29^**^ | 1 |  |  |
| Gender | 0.02 | 0.04 | -0.11^**^ | 0.11^**^ | -0.06^*^ | -0.06^*^ | 0.03 | -0.07^**^ | -0.11^**^ | -0.10^**^ | 1 |  |
| Age | -0.03 | -0.06^**^ | -0.16^**^ | -0.09^**^ | -0.15^**^ | -0.08^**^ | -0.16^**^ | -0.03 | -0.06^*^ | -0.04 | 0.07^*^ | 1 |

*^a.^* In the correlation analysis, fruit eating, vegetable eating, beverage drinking, physical activity, screen time during weekdays, screen time during weekends, sleep duration during weekdays, sleep duration during weekends, smoking, alcohol drinking and sex were categorical variables. Age was a continuous variable. FE = fruit eating, VE= vegetable eating, BD = beverage drinking, MVPA = moderate to vigorous physical activity, STD = screen time (weekdays), STE = screen time (weekends), SDD = sleep duration (weekdays), SDE = sleep duration (weekends), SM = smoking, AD = alcohol drinking. * *p* <0.05, * **p* < 0.001.
